# A Rare Case of Dermatofibrosarcoma Protuberans of the Thumb in an 80-year-old Woman

**DOI:** 10.7759/cureus.2016

**Published:** 2018-01-02

**Authors:** Georgi P Georgiev, Svetoslav A Slavchev, Julian Ananiev

**Affiliations:** 1 Department of Orthopaedics and Traumatology, Medical University of Sofia, Bulgaria, University Hospital Queen Giovanna; 2 Department of Orthopaedics and Traumatology, Medical University of Sofia, Bulgaria, University Hospital of Orthopaedics - Sofia; 3 Pathology, Medical Faculty Trakia University

**Keywords:** dermatofibrosarcoma protuberans, thumb, surgery

## Abstract

Dermatofibrosarcoma protuberans (DFSP) has been described as a rare, locally invasive, malignant fibroblastic tumor with a high rate of recurrence that usually affects middle-aged patients. Herein, we describe a rare case of a pedunculated dermatofibrosarcoma protuberans of the right thumb in an 80-year-old woman treated with excision through the base of the pedicle. We also make a brief literature review concerning this tumor.

## Introduction

Dermatofibrosarcoma protuberans (DFSP) has been described as a rare, slowly growing, low-grade malignancy that is locally aggressive and has a high rate of recurrence and low metastatic potential [1]. Its incidence is around four per million person-years, and it usually affects adults between 20 and 59 years of age with a slight male preponderance. DFSP is rarely encountered in children and elderly persons. Several cases of congenital variants of DFSP have also been described [2]. Most commonly DFSP affects the trunk (40%-50%), followed by the proximal segments of the upper and lower limbs (30%-40%) and the head and neck region (10%-15%) [3]. There are only a few cases reports of DFSP in the fingers but not in an elderly patient [1].

Herein, we present a rare case of a pedunculated DFSP of the thumb in an 80-year-old woman.

## Case presentation

**Figure 1:**
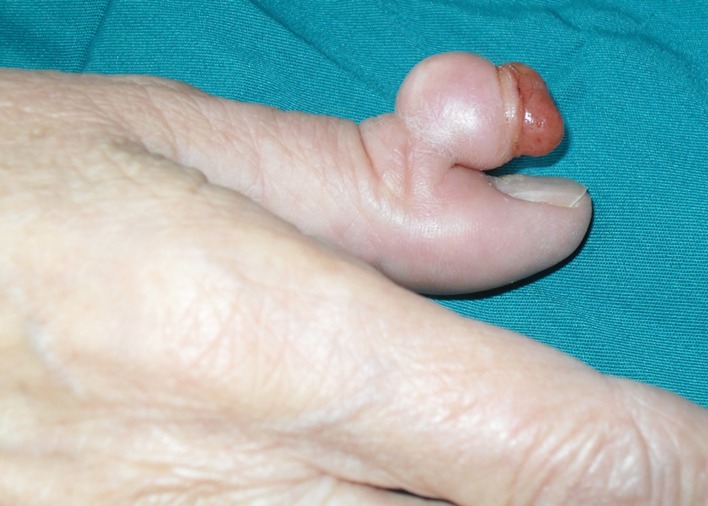
Preoperative photograph of a pedunculated DFSP DFSP: dermatofibrosarcoma protuberans

An 80-year-old Caucasian woman presented to the University Hospital of Orthopaedics with a formation in her right thumb with a duration of more than 10 years. A few months prior to presentation, the formation had gradually ulcerated with no bleeding, secretion, or pain. A physical examination revealed a non-tender, rounded, pedunculated soft-tissue mass measuring about one centimeter in diameter on the dorsal aspect of the interphalangeal joint of the thumb. The skin over the distal third of the mass was completely missing, revealing the underlying, slightly exsiccated, homogeneous yellowish tumor surface (Figure 1).

**Figure 2:**
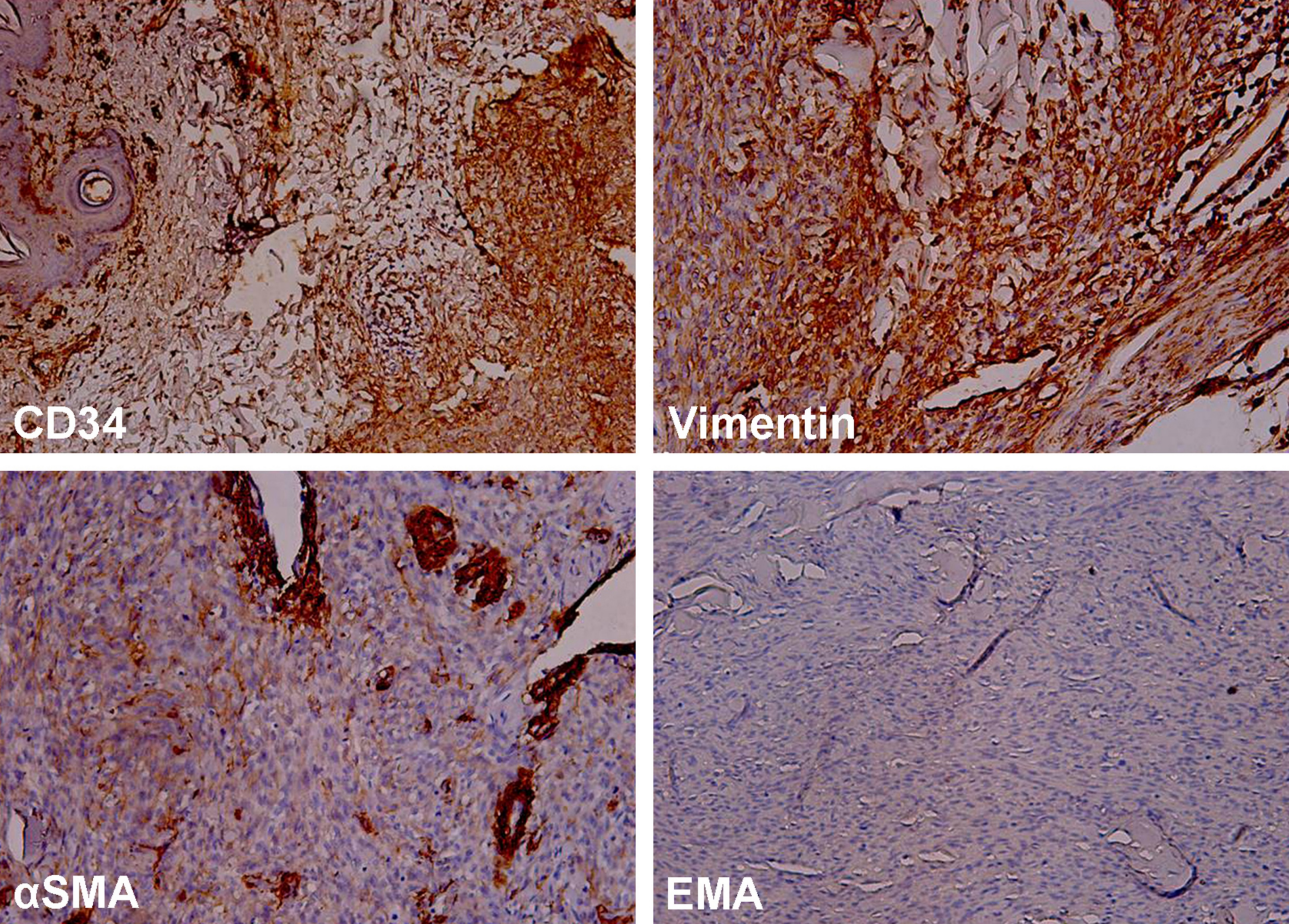
An immunohistochemical examination of DFSP revealed a strong expression (brown-red color) of CD34 and vimentin, positivity (brown-red color) for α-SMA in blood vessel walls and single cells, and a negative reaction for EMA. DFSP: dermatofibrosarcoma protuberans; α-SMA: α-smooth muscle actin; EMA: epithelial membrane antigen

The pedicle was about five millimeters wide, and its skin had a normal, gross appearance. The function of the thumb was not affected. Laboratory tests were within normal limits. The lesion was excised through the base of the pedicle, and the surgical wound healed uneventfully. Upon a histological examination, dermatofibrosarcoma protuberans was diagnosed with areas of the dermal proliferation of bland monomorphic spindle cells arranged in whorled and storiform patterns and with no significant nuclear atypia, mitoses, or necrosis. An immunohistochemical examination revealed a strong expression of CD34 and vimentin, and positivity for α-smooth muscle actin (α-SMA) in blood vessel walls and single cells. The tumor cells were negative for epithelial membrane antigen (EMA) (Figure 2).

No local recurrence was detected during a follow-up of two years. Later, the patient died of an unrelated condition.

## Discussion

DFSP has been described as the most common dermal sarcoma. Morphologically, DFSP is composed of spindle-shaped cells with a diffuse CD34 expression. These cells arise in the dermis and could also disseminate in the subcutaneous fat. At the ultrastructural level, these cells resemble fibroblasts and are similar to dermal dendrocytes [3].

This malignant fibroblastic tumor is characterized by a specific chromosomal translocation t(17;22)(q22;q13) in 90% of cases, which leads to fusion of the platelet-derived growth factor beta (PDGFbeta) gene with the collagen type 1 alpha 1 (COL1alpha1) gene [2-3].

Morphologically, in a differential diagnosis, DFSP could be mistaken for dermatofibroma, peripheral nerve sheath tumors (e.g., neurofibroma or schwannoma), malignant melanoma, and morpheaform basal cell carcinoma [2]. It should be also noted that apart from DFSP, other tumors, such as epithelioid sarcoma, spindle cell lipoma, CD34-positive cellular digital fibromas, and solitary fibrous tumors, could also express CD34 [4].

DFSPs usually appear as nodules, plaques, or papules and could affect any part of the body [5]. The color of this tumor could vary from brown to reddish-blue to violaceous. Usually, DFSP has a long history of slow growth. In some cases, especially if recurrent or of long duration, this tumor could affect deeper tissues, such as fascia, muscle, periosteum, and even bone. Rapid growth may be due to fibrosarcomatous transformation [3]. According to Criscione and Weinstock [4], when affecting the extremities or the head and neck region, DFSP is more invasive compared to trunk locations. Rarely, lung metastases develop, especially after multiple recurrences [4]. Lymph node metastases have also been described [6]. The reported five-year survival rate is 99.2% [4].

After an extensive review of the PubMed database, we identified four similar cases published in the literature, but not in an elderly patient. Campos, et al. [7] presented a case of myxoid DFSP on the middle finger of the left hand of a 14-year-old girl. Chiang, et al. [1] reported a case of DFSP of the left middle finger in a 29-year-old pilot treated with a series of re-excisions and skin grafting. Atkinson, et al. [8] presented a case with DFSP of the non-dominant ring finger of a 31-year-old police officer treated with metacarpophalangeal disarticulation. Reimann and Fletcher [9] described DFSP of the left index finger in a 54-year-old female treated with three consecutive excisions within two years.

To the best of our knowledge, this is the first report of a pedunculated DFSP of the hand.

Although nonspecific, imaging studies, such as magnetic resonance imaging (MRI), computed tomography (CT), and ultrasound, are essential for the preoperative evaluation of this tumor [2-3].

The gold standard for the treatment of DFSP is adequate surgical removal. Inadequate treatment leads to high recurrence rates (20% to 50%) [3,5]. The median time of recurrence has been reported as 32 months after surgery. Therefore, a long time of follow-up is crucial [3].

In the current literature data, there are different strategies considering the width of the surgical margin and the excision techniques. Some authors prefer Mohs micrographic surgery (MMS) or slow MMS, but others recommend wide excision. In the latter, the surgical margins should be at least three centimeters wide to reduce the recurrence rate [2]. However, that might not always be possible, especially in the region of the fingers, as in our case. After the removal of this tumor, primary skin closure, skin grafts, local tissue rearrangement, pedicle flaps, or free flaps for covering the tissue defect could be performed [10].

It is well-established that chemotherapy has a palliative effect. Target therapy with Imatinib has mixed results [3]. Radiotherapy is also used in inoperable cases [2].

## Conclusions

In conclusion, we described an extremely rare case of a pedunculated DFSP of the thumb in an 80-year-old lady. Commonly, the finger localization of DFSP is associated with a high rate of recurrence. Therefore, it is essential for patients with DFSP of the hand to be evaluated by a multidisciplinary team, including a hand surgeon and an experienced pathologist, in order to achieve recurrence-free outcomes and good hand function.
